# An improvised one-step sucrose cushion ultracentrifugation method for exosome isolation from culture supernatants of mesenchymal stem cells

**DOI:** 10.1186/s13287-018-0923-0

**Published:** 2018-07-04

**Authors:** Suchi Gupta, Sonali Rawat, Vivek Arora, Sarat Kumar Kottarath, Amit Kumar Dinda, Pradeep Kumar Vaishnav, Baibaswata Nayak, Sujata Mohanty

**Affiliations:** 1 Stem Cell Facility, (DBT-Centre of Excellence for Stem Cell Research), All India Institute of Medical Sciences, New Delhi, 110029 India; 2 Department of Pathology, All India Institute of Medical Sciences, New Delhi, India; 3 Sophisticated Advanced Instrument Facility, All India Institute of Medical Sciences, New Delhi, India; 4 Department of Gastroenterology and Human Nutrition Unit, All India Institute of Medical Sciences, New Delhi, India

**Keywords:** Mesenchymal stem cells, Exosomes, Sucrose cushion ultracentrifugation

## Abstract

**Background:**

Exosomes are nanovesicles (30–120 nm) of endosomal origin. These exosomes contain various functional proteins and RNAs that could be used for therapeutic purposes. Currently, having a standard method for exosome isolation retaining its biological properties with increased yield and purity is a major challenge. The most commonly used method is differential ultracentrifugation but it has its own disadvantages, which include high time consumption, low yield due to disruption of exosome integrity, and high protein contaminants. In this study, we have identified an improved method addressing these problems for exosome isolation using ultracentrifugation since it is cost-effective and used worldwide.

**Method:**

We have compared differential ultracentrifugation with the modified method called one-step sucrose cushion ultracentrifugation for exosome isolation. The conditioned serum-free media from human mesenchymal stem cells cultured for 48 h was collected for exosome isolation. The cellular debris was removed by centrifugation at 300*g* for 10 min, followed by centrifugation at 10,000*g* for 30 min to remove microvesicles. Equal volumes of pre-processed conditioned media were used for exosome isolation by direct ultracentrifugation and one-step sucrose cushion ultracentrifugation. The exosomes isolated using these methods were characterized for their size, morphology, concentration, and surface marker protein expression.

**Result:**

It was observed that the recovery of exosomes with cup-shaped morphology from one-step sucrose cushion ultracentrifugation was comparatively high as estimated by nanoparticle tracking analysis and electron microscopy. These results were confirmed by Western blotting and flow cytometry.

**Conclusion:**

We conclude that this one-step sucrose cushion ultracentrifugation method provides an effective and reproducible potential standard method which could be used for various starting materials for isolating exosomes. We believe that this method will have a wide application in the field of extracellular vesicle research where exosome isolation with high yield and purity is an imperative step.

**Graphical abstract:**

*Schematic representation of comparison of UC and SUC exosome isolation methods for tissue-specific human mesenchymal stem cells. The SUC isolation method yields a greater number of cup-shaped exosomes with a relatively homogenous population for mass-scale production of exosomes for downstream analysis*. *Abbreviations: SUC One-step sucrose cushion ultracentrifugation, UC Direct ultracentrifugation.*
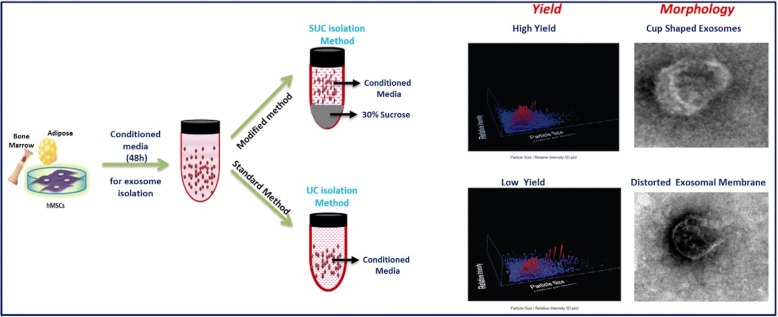

**Electronic supplementary material:**

The online version of this article (10.1186/s13287-018-0923-0) contains supplementary material, which is available to authorized users.

## Background

Mesenchymal stem cells (MSCs) are multi-potent self-renewing cells having remarkable potential in regenerative medicine. These MSCs used in clinical trials for regeneration studies are derived from various sources like bone marrow (BMSCs), dental pulp, adipose tissue (ADSCs), and Wharton’s jelly [1–3]. Initially, the mechanisms relevant to their biological functions such as homing, transdifferentiation, and immunomodulation have been reported [1–4]. However, recently, it has been demonstrated that the paracrine action of MSCs plays a significant role in repair and regeneration through secretion of extracellular vesicles called exosomes. The exosomes are small (30- to 120-nm) nanovesicles originating from endosomes and have been shown to elicit similar biological activity as stem cells themselves [5, 6]. These, MSC-derived exosomes are becoming a new beacon for cell free therapy, having low risk of immune rejection to allogeneic administration [7]. Thus, isolation of exosomes retaining their biological activity is a major challenge because of their small size and labile nature. The different methods of exosome isolation are differential ultracentrifugation, size exclusion chromatography, immune affinity, and so on. But the most commonly used method for isolation of exosomes is differential ultracentrifugation because of its ease of handling large volumes of conditioned media, thereby improving the yield. However, there are contraindicated reports for ultracentrifugation-based methods of exosome isolation for low yield and purity, loss of membrane integrity, and several other parameters [8–10].

The most commonly used approach is a two-step isolation method which includes direct ultracentrifugation (UC) followed by 30% sucrose density ultracentrifugation to increase purity by removing protein contamination [11]. The density of sucrose (1.12 to 1.18 g/mL) is equivalent to that of exosomes (1.15 to 1.19 g/mL) which produces a cushioning effect maintaining integrity of exosomes and separating protein contaminants of high density (1.22 g/mL) [12].

**Fig. 1:**
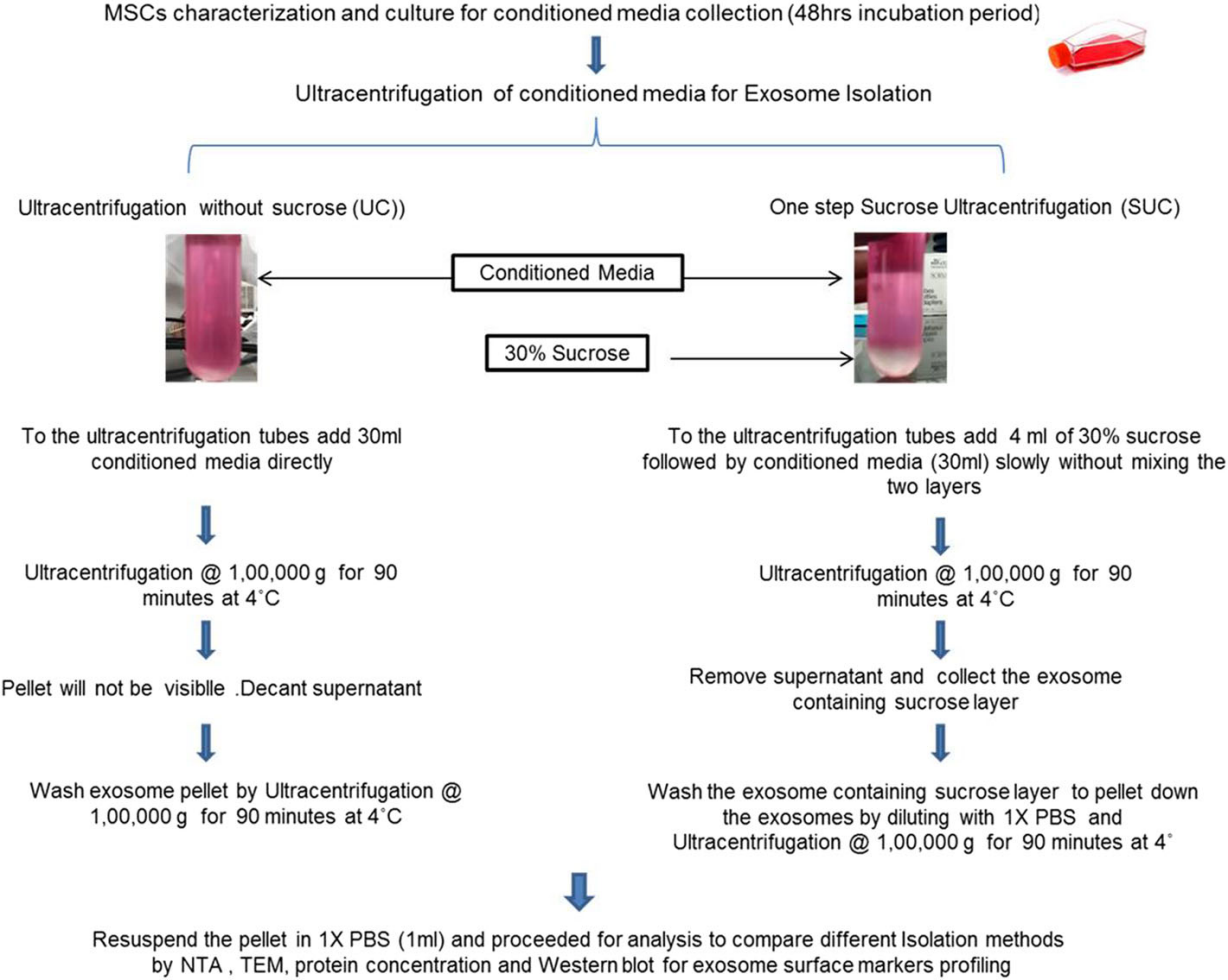
Schematic representation of exosome isolation using standard method (direct ultracentrifugation) and modified method (one-step sucrose cushion ultracentrifugation). Abbreviations: *MSC* mesenchymal stem cell, *NTA* Nanoparticle Tracking Analysis, *PBS* phosphate-buffered saline, *TEM* transmission electron microscopy

In the present study based on density and cushioning property of sucrose, we have developed a modified one-step sucrose cushion ultracentrifugation (SUC) method for isolation of exosomes and compared it with UC for better yield, exosome integrity, and purity from protein contaminants. The exosomes isolated were characterized for their size, morphology, yield, and surface marker protein expression. This method is different from the previously published methods, in which pre-enriched exosomes isolated by UC were further purified by 30% sucrose density ultracentrifugation. It was assumed that low yield of exosomes from UC would be further reduced when processed for a second step using sucrose cushion ultracentrifugation. Therefore, we have compared the modified method with the UC step only. Figure 1 shows a schematic representation of the exosome isolation method using ultracentrifugation adopted for exosome isolation comparison from human tissue-specific MSCs (hMSCs).

## Materials and methods

### Revival, expansion, and characterization of cryopreserved human mesenchymal stem cells

MSCs used in this study were isolated from donors with consent after obtaining ethical clearance (ref. no. ICSCR/34/15(R)) from the Institutional Committee for Stem Cell Research, All India Institute of Medical Science, New Delhi, India. Bone marrow– and adipose tissue–derived hMSCs, obtained from three donors each and cryopreserved during previous projects in liquid nitrogen, were used.

Cryopreserved BMSCs and ADSCs were revived and propagated in Dulbecco’s modified Eagle’s medium–low glucose (DMEM-LG) media (Life Technologies, Carlsbad, CA, USA) containing 10% fetal bovine serum (FBS) (HyClone, part of Thermo Fisher Scientific, Waltham, MA, USA), 2 mM L-glutamine, 100 U/mL of penicillin, and 100 U/mL of streptomycin (Life Technologies). These cells were subcultured at 70% confluence. For enrichment of exosomes, these hMSCs were propagated in serum-free media (STEMPRO^®^ MSC SFM CTS, Thermo Fisher Scientific) for 48 h. The hMSCs were stained for tri-lineage (adipocytes, osteocytes, and chondrocytes) and specific surface markers for flow cytometry which include CD105, CD73, CD29 and CD90, HLA-I, CD34/45, and HLA class II. Selection of these markers and flow cytometry were carried out in accordance with described protocols [13, 14].

### Isolation of human mesenchymal stem cell–derived exosomes

The conditioned serum-free media from hMSCs cultured for 48 h was pooled together for exosome isolation. The cellular debris was removed by centrifugation at 300*g* for 10 min, followed by centrifugation at 10,000*g* for 30 min to remove microvesicles. Equal volumes of pre-processed conditioned media were used for exosome isolation by UC and one-step SUC. For the UC method, conditioned media was directly centrifuged at 1,00,000*g*, 4 °C for 90 min using Sorvall™ WX 90+ ultracentrifuge in swinging Bucket rotor (Thermo Fisher Scientific). For SUC-based isolation, conditioned media was loaded slowly over 4 mL of 30% sucrose solution (prepared in 1 × phosphate-buffered saline, or PBS), forming a layer, and centrifuged at 1,00,000*g*, 4 °C for 90 min using Sorvall™ WX 90+ ultracentrifuge in swinging Bucket rotor (Thermo Fisher Scientific). For the UC method, the supernatant was discarded and the exosome pellet was resuspended in 1× PBS and washed by ultracentrifugation at 1, 00,000*g* at 4 °C for 90 min. On the other hand, for the SUC method, the supernatant was discarded and the sucrose layer (~5 mL) was resuspended in 1 × PBS and ultracentrifuged at 1, 00,000*g* at 4 °C for 90 min to pellet down the exosomes. After this, the exosomes were resuspended in 500 μL 1 × PBS and stored at − 80 °C for further use.

### Characterization of exosomes

#### Nanoparticle tracking analysis

The exosomes were diluted (1:10) in 1× PBS for nanoparticle tracking analysis (NTA) by NanoSight LM20 (NanoSight, Malvern Panalytical Ltd, Malvern, UK). The Brownian motion of each particle was tracked between frames and the size was calculated by using the Stokes-Einstein equation.

#### Transmission electron microscopy

The exosome suspension (5 μL of undiluted and diluted 1:1000 in 1 × PBS) was placed on Formvar-carbon–coated copper grids and allowed to adsorb for 5 min in a dry environment. The grids were washed in drops of 1× PBS and stained with 2% phosphotungstic acid solution for 1 min. Thereafter, grids were air-dried and were observed under the electron microscope (Tecnai, FEI, Hillsboro, OR, USA). For morphometric analyses of exosome for size, shape, and number, three images per sample were taken and analyses were carried out individually by three individuals for unbiased results.

#### Western blotting

Exosomes (the starting volume was kept constant for both UC and SUC method comparison) were lysed in RIPA buffer, 1 mM phenylmethylsulfonyl fluoride (PMSF), and protease inhibitors. The protein concentration of lysates was measured by using a bicinchoninic acid assay (BCA) protein assay kit (Pierce, part of Thermo Fisher Scientific). Equal volumes of exosomal lysates (50 μL) were subjected to 12.5% SDS-PAGE in non-reducing conditions for CD63 and reducing conditions for Alix and transferred to a polyvinylidene difluoride (PVDF) membrane (MDI Membrane Technologies, Harrisburg, PA, USA) by using a wet transfer system (Bio-Rad Laboratories, Hercules, CA, USA). The blot was blocked in 5% non-fat skimmed milk in 1 × TBS-T solution followed by incubation in CD63 primary antibody (1:5000; Abcam, Cambridge, MA, USA), Alix primary antibody (1:500; Santa Cruz Biotechnology, Dallas, TX, USA), and GAPDH (1:3000; Genetix, San Jose, CA, USA) overnight at 4 °C. The blot was washed and incubated with horseradish peroxidase (HRP)-conjugated secondary antibody and developed by using an ECL imager (Invitrogen, a brand of Thermo Fisher Scientific).

#### Flow cytometry of exosomes

Exosome surface marker profiling by flow cytometry was carried out by using CD63 tagged magnetic beads (Life Technologies). Briefly, exosomes (100 μL) were incubated with 1×10^5^ numbers of CD63 tagged magnetic beads (10 μL) overnight at 4 °C on an orbital shaker. CD63 magnetic beads bound to exosomes were further purified by using a magnetic-activated cell sorting (MACS) magnetic separator (Miltenyi Biotec, Bergisch Gladbach, Germany). These purified exosome-bound beads were further incubated with PKH26 dye (Sigma-Aldrich, St. Louis, MO, USA) at room temperature for 40 min on the shaker and washed twice by using isolation buffer (1× PBS 0.1% bovine serum albumin [BSA]). These CD63 tagged, PKH26 dye stained exosome-bound magnetic beads and isotype control beads were acquired by flow cytometry (Becton Dickinson, Franklin Lakes, NJ, USA) and analyzed with FACSDiva software (version 6.1.3). Apart from the exosome-specific marker, CD63, parent cell surface marker (MSC-specific marker) expression on exosomes was evaluated by using flow cytometry. For this, exosomes (100 μL) were incubated with 1×10^5^ CD63 tagged magnetic beads (10 μL) overnight at 4 °C on the orbital shaker. CD63 magnetic beads bound to exosomes were further purified by using the MACS magnetic separator. To these bead-bound exosomes, monoclonal antibodies specific for CD105-APC, CD73-PE, CD29-FITC, CD90-PerCp-Cy5.5, HLA-ABC-APC, and HLA-DR-FITC (BD Pharmingen, San Diego, CA, USA) were added and incubated with intermediate mixing at room temperature for 40 min on the shaker and washed twice by using isolation buffer (1× PBS 0.1% BSA). Isotype control beads for all of these tagged antibodies were acquired by flow cytometry and analyzed with FACSDiva software (version 6.1.3).

### Statistical analysis

In this study, all the data analysis was carried out by using Graph Pad Prism 5 software (GraphPad Software, La Jolla, CA, USA). *P* values were calculated by using Student’s *t* test. All the data were expressed as mean ± standard error of the mean from *n* = 3 for each tissue source. For all analyses, differences with a *P* value of less than 0.05 were considered statistically significant.

## Results

### Characterization of mesenchymal stem cells and their differentiation potential

**Fig. 2:**
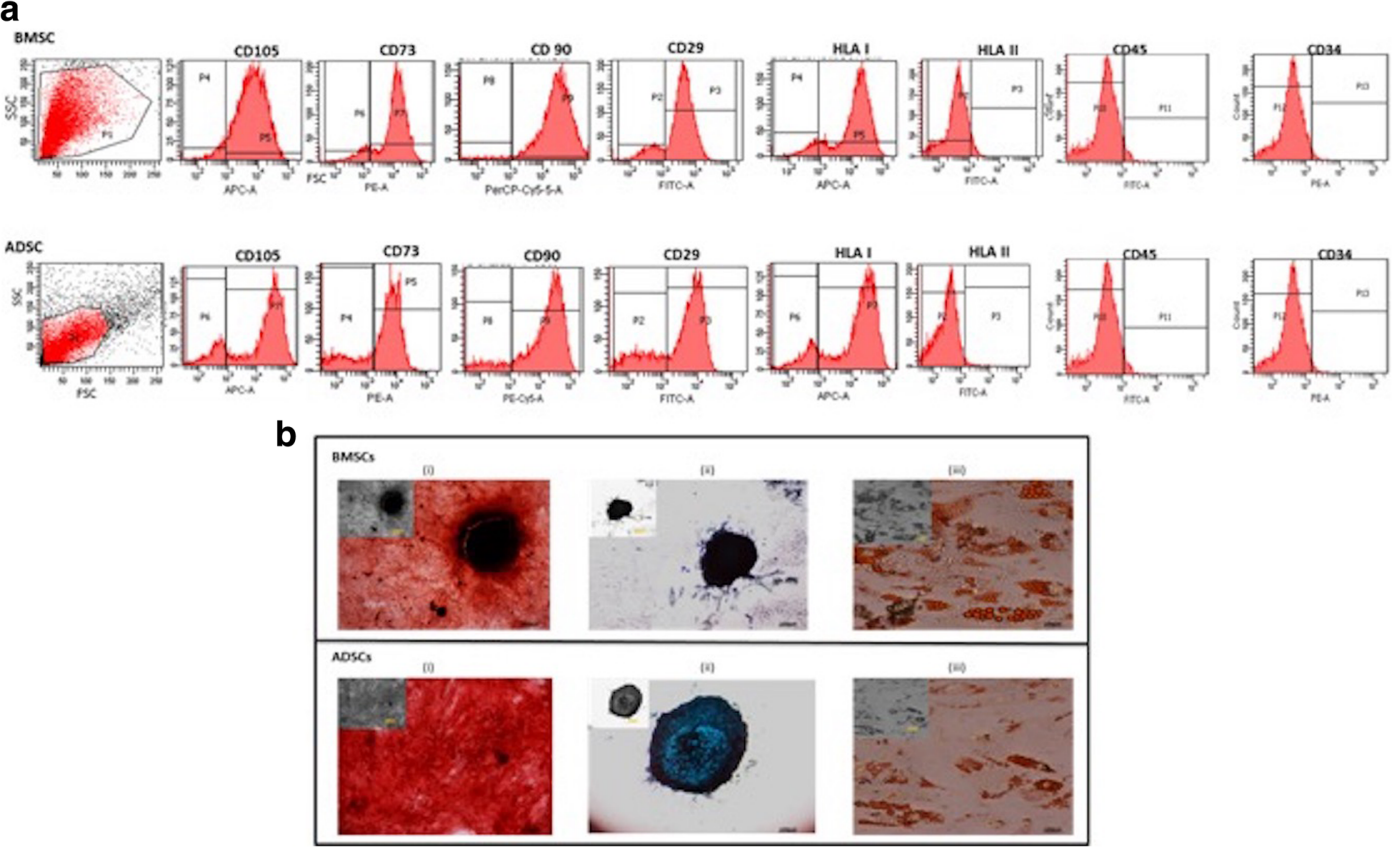
Characterization of BMSCs and ADSCs using **(a)** flow cytometry for positive markers such as CD29, CD73, CD90, CD105, and HLA-I and negative markers such as HLA-II, CD34, and CD45. **b** Tri-lineage differentiation potential. (i) Osteogenesis was determined by Alizarin red S staining of the extracellular mineralized matrix. (ii) Adipogenesis was determined by using Oil red O staining of lipid droplets. (iii) Chondrogenesis was determined by Alcian blue staining of proteoglycan. All images were taken at 20× magnification. Abbreviations: *ADSC* adipose tissue–derived mesenchymal stem cell, *BMSC* bone marrow–derived mesenchymal stem cell

MSCs isolated from bone marrow and adipose tissue of adult donors (18–35 years old) that were cryopreserved during previous studies were used. These hMSCs were characterized by flow cytometry for surface staining of hMSCs surface markers. These hMSCs were found to be positive for CD29, CD73, CD90, CD105, and HLA I and negative for HLA II and hematological markers, CD34/45 (Fig. 2a). Tri-lineage differentiation potential of these hMSCs was evaluated by staining with Alizarin red, Oil red O, and Alcian blue for osteocytic, adipocytic, and chondrocytic differentiation, respectively (Fig. 2b).

### Comparative evaluation of exosome size and numbers by nanoparticle tracking analyses

**Fig. 3:**
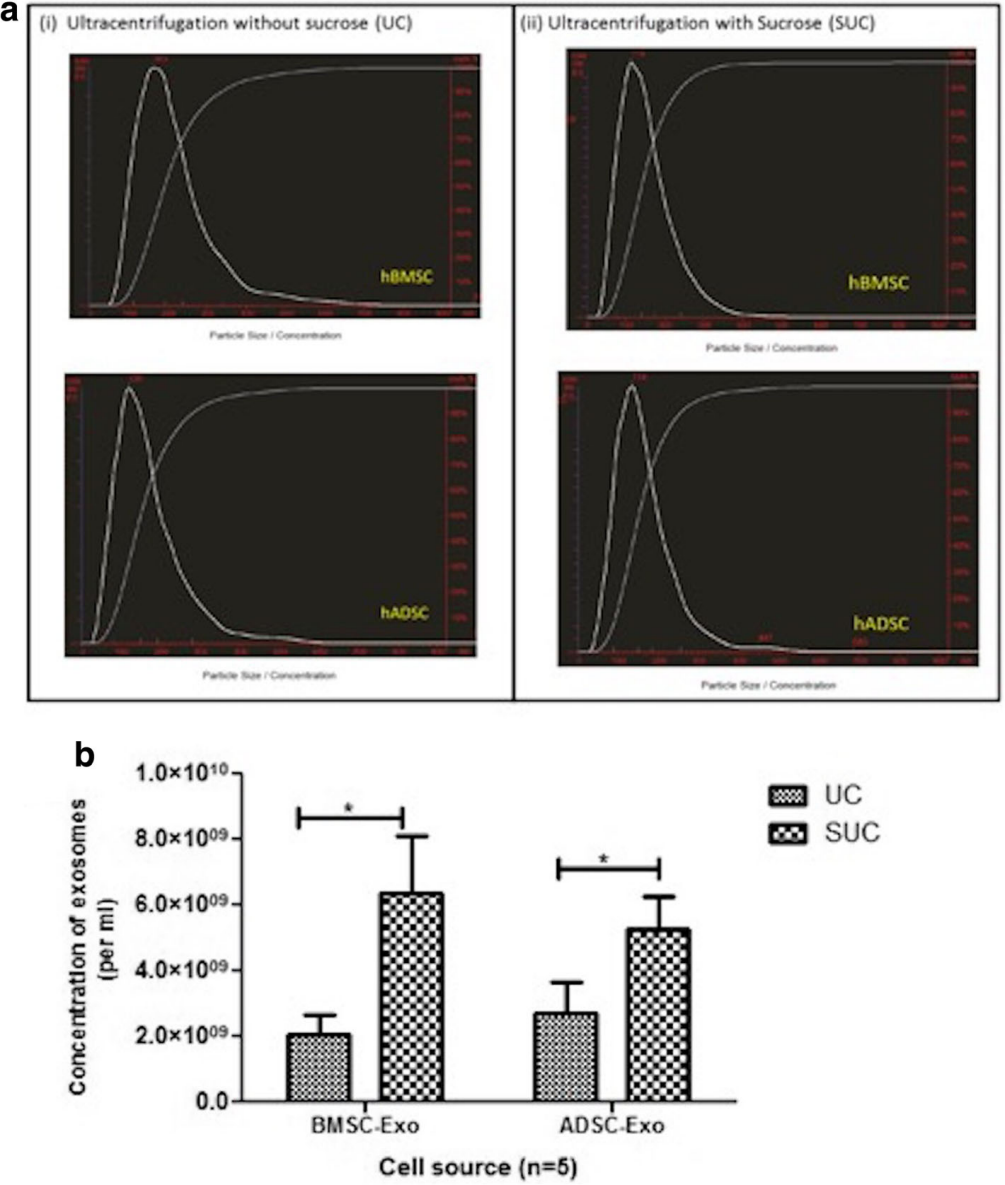
Size distribution and concentration of hMSC-derived exosomes. **a** As measured by Nanoparticle Tracking Analyses (NTA), particle size for hMSC exosomes isolated by both methods was within the range of 30 to 120 nm. Size distribution and concentration of hMSC-derived exosomes as measured by NTA. **b** Representative graph plots depicting the significant increase in the yield of exosomes isolated by using the modified method (SUC). Results are mean ± standard error of the mean of three independent experiments. *Significant with *P* value of less than 0.05. Abbreviations: *ADSC* adipose tissue–derived mesenchymal stem cell, *BMSC* bone marrow–derived mesenchymal stem cell, *hMSC* human mesenchymal stem cell, *ns* non-significant, *SUC* one-step sucrose cushion ultracentrifugation, *UC* direct ultracentrifugation

Both ADSCs and BMSCs were purified by UC and SUC. The size of exosomes isolated by both the methods was within the expected size range of 30–120 nm (Fig. 3a). A statistically significant increase in concentration of exosomes was observed in the SUC in comparison with the UC method (Fig. 3b). For BMSCs, exosome concentrations were 2 × 10^9^ particles per milliliter and 7 × 10^9^ particles per milliliter for UC and SUC, respectively. For ADSCs, concentrations were 3.06 × 10^9^ particles per milliliter and 5.60 × 10^9^ particles per milliliter for UC and SUC method, respectively.

### Morphometric analyses of exosomes by transmission electron microscopy

**Fig. 4:**
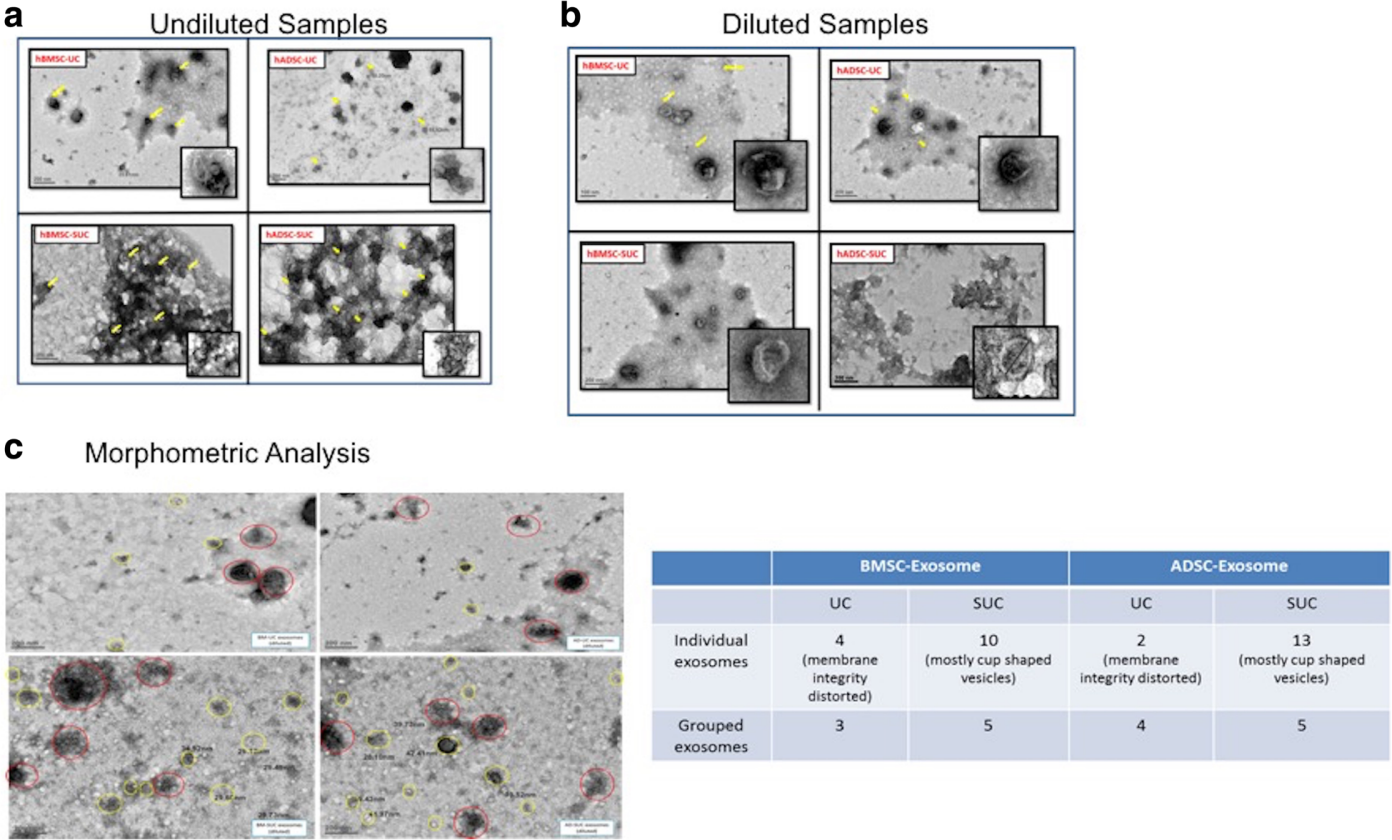
Transmission electron microscopic (TEM) pictures of exosomes isolated by hMSCs by using the UC and SUC methods. **a** Representative images depicting a higher number of exosomes in the SUC method for both tissuespecific hMSCs. **b** & **c** Morphometric evaluation of exosome by further diluting the samples for shape, size, and number. Exosomes isolated by the SUC method were cup-shaped and greater in number compared with those isolated by the UC method. Also, small-sized vesicles (<30 nm) were observed to be in clusters of four to five vesicles each. Red circle depicts grouped or coupled vesicles Yellow circle depicts individual vesicles. All images were taken at 15,000× magnification and for morphological analyses images were magnified at 20,000× magnification. Abbreviations: *hADSC* human adipose tissue–derived mesenchymal stem cell, *hBMSC* human bone marrow–derived mesenchymal stem cell, *hMSC* human mesenchymal stem cell, *ns* non-significant, *SUC* one-step sucrose cushion ultracentrifugation, *UC* direct ultracentrifugation

In transmission electron microscopy (TEM), cup-shaped vesicles were observed (Fig. 4), which corroborate to cup-shaped morphology of exosomes. The size of these exosomes was within the range of 30 to 120 nm. The number of exosomes observed in undiluted stained samples of SUC-based isolation was much higher than UC-based isolation method for both BMSC- and ADSC-derived exosomes, as can be seen in the TEM images. There was clustering of exosomes in undiluted samples; therefore, these exosomes were diluted in a 1:1000 ratio to evaluate their morphology. In the SUC method, exosomes were cup-shaped and maintained their integrity when compared with UC-isolated exosomes, which were very low in number, and membrane was observed to be distorted. However, morphometric analyses for exosomes isolated by both methods were carried out and data were quantified by counting the number of vesicles per image by three individuals (Fig. 4c). It was observed that exosomes isolated by the SUC method were cup-shaped and greater in number compared with those isolated by the UC method where membrane integrity of exosomes was disrupted. Small-sized vesicles (<30 nm) were also observed to be in clusters of four to five vesicles each. A central depression, which is a characteristic for exosomes under TEM, was also observed.

### Confirmation of exosomes by Western blot and flow cytometry using exosome-specific surface marker

**Fig. 5:**
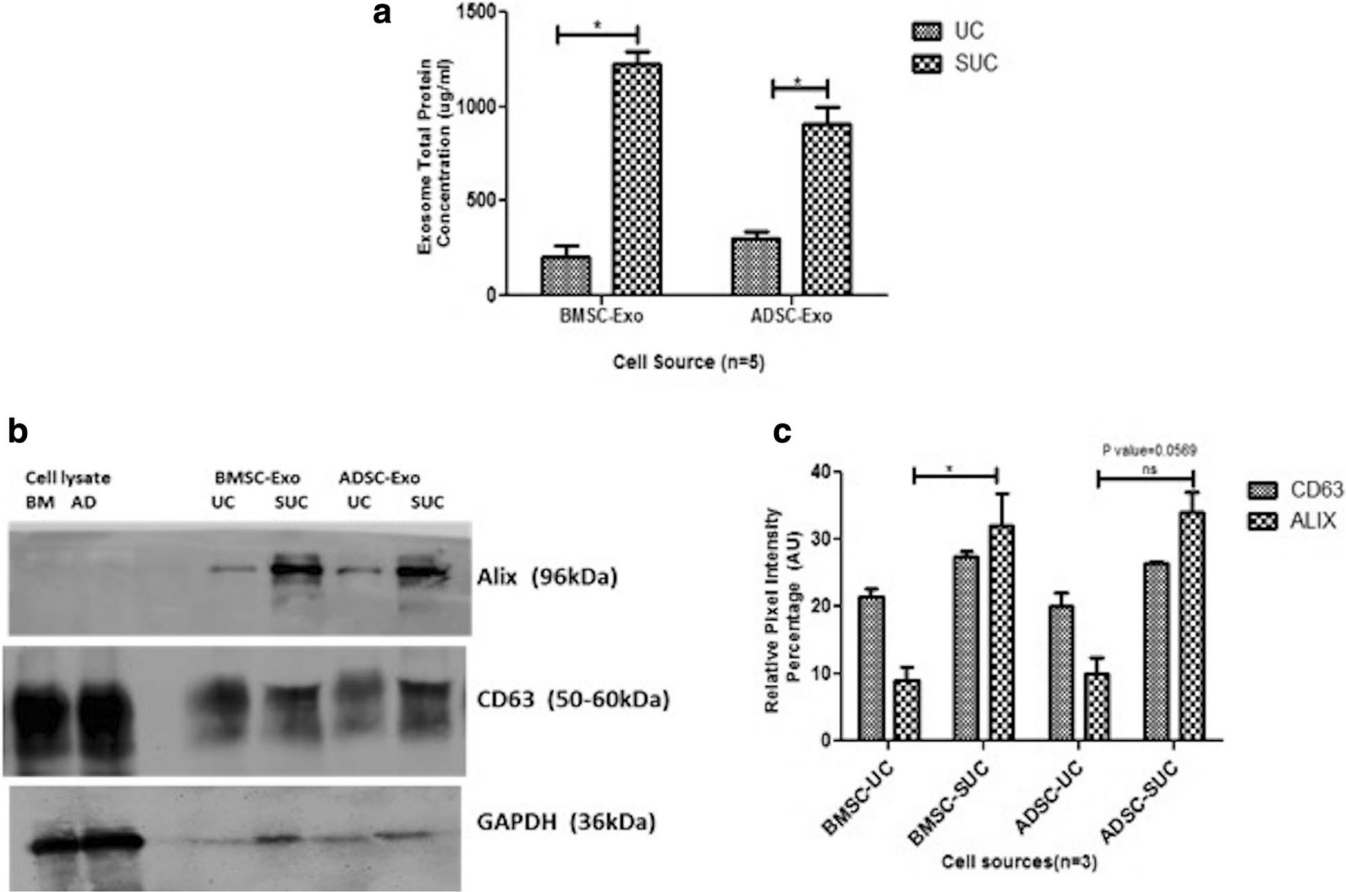
Surface marker profiling of exosomes by Western blotting. **a** Total protein concentration of exosomes by bicinchoninic acid assay (BCA): Representative graph plots depict the comparison of total protein concentration in exosomes which showed that the concentration of exosomal protein was significantly higher for the SUC isolation method. **b** Western blot for CD63 and Alix protein expression in exosomes (50 μL of total exosomal protein was loaded). GAPDH was used as a housekeeping gene. Unlike CD63, Alix was expressed specifically in exosomes. **c** Densitometric analysis of CD63 and Alix expression in exosomes showed a higher protein expression for the SUC isolation method. Results are mean ± standard error of the mean of three independent experiments. *Significant with *P* value of less than 0.05. Abbreviations: *AD* adipose tissue, *ADSC* adipose tissue–derived mesenchymal stem cell, *AU* arbitrary unit, *BM* bone marrow, *BMSC* bone marrow–derived mesenchymal stem cell, *ns* non-significant, *SUC* one-step sucrose cushion ultracentrifugation, *UC* direct ultracentrifugation

The isolated exosomes derived from BMSCs and ADSCs were further confirmed to be exosomes by immunoblotting and flow cytometry by using antibody specific to exosome surface marker CD63. Protein concentration was high for exosomes isolated using SUC than UC method for both ADSCs and BMSCs samples by colorimetric assay by using a BCA assay kit (Fig. 5a). In Western blot, a CD63 protein–specific band (~55 to 30 kDa) and an Alix protein–specific band (~96 kDa) were observed in lysate of exosome isolated by the UC and SUC methods (Fig. 5b). Densitometry analysis indicated a higher concentration of exosomal protein in the SUC than the UC method (Fig. 5c). GAPDH was used as an internal reference control. We loaded equal volumes of eluted exosome to observe variation in exosome yield; a higher yield was observed in SUC as compared with UC. The densitometric ratio was also calculated and was higher for the SUC method [CD3/GAPDH 2.93, 2.65; Alix/GAPDH 0.46, 0.59 for BM and AD-MSC] than the UC method [CD3/GAPDH 2.78, 2.41; Alix/GAPDH 0.18, 0.17 for BM and AD-MSC]. The smear pattern of CD63 is due to the nature (glysosylated) of this protein.

**Fig. 6:**
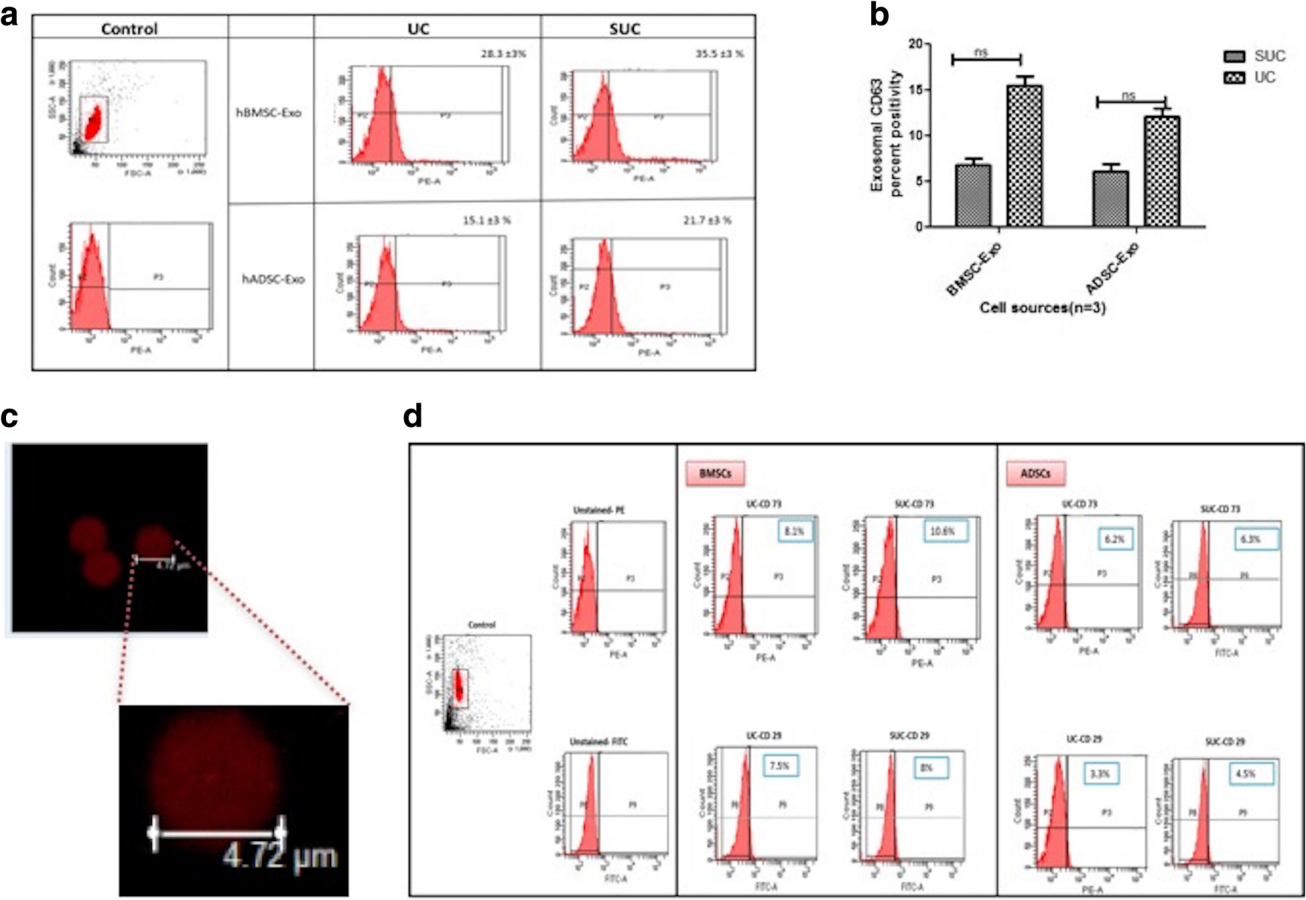
**a** Representative flow cytometry histogram plots for CD63-positive exosomes isolated by both methods and (**b**) its quantification. **c** Confocal microscopy (Leica, Wetzlar, Germany) was used to visualize the PKH26-labeled exosomes bound to magnetic beads (magnification 63×). **d** Representative flow cytometry histogram plots for BMSC- and ADSC-derived exosomes to analyze the expression of parent cell surface markers (CD73and CD29). Results are mean ± standard error of the mean of three independent experiments. *Significant with *P* value of less than 0.05. Abbreviations: *ADSC* adipose tissue–derived mesenchymal stem cell, *BMSC* bone marrow–derived mesenchymal stem cell, *ns* non-significant, *SUC* one-step sucrose cushion ultracentrifugation, *UC* direct ultracentrifugation

The CD63-positive exosomes were quantified by flow cytometry. For BMSCs, 35.5 ± 3% exosomes were CD63-positive when isolated using the SUC method in comparison with 28.3 ± 3% CD63-positive exosomes isolated by using the UC method. For ADSCs, 21 ± 3% CD63-positive exosomes were isolated by using the SUC method in comparison with the UC method where CD63-positive exosomes percentage was 15.1 ± 3% (Fig. 6a). There was no significant increase in CD63-positive exosomal population isolated by either method (Fig. 6b). However, it could be seen that the yield of these exosomes has improved. Also, to further confirm the exosome binding to these magnetic beads, these PKH26-labeled exosome-bound magnetic beads were visualized by confocal microscopy as seen in Fig. 6c. The CD63-bound exosomes were further evaluated by flow cytometry for surface expression of other hMSC markers by staining with dye-labeled monoclonal antibodies. The detectable level of surface expression in the exosomes was observed for surface markers CD73 and CD29. However, the level of expressions for other hMSCs markers (CD105, CD90, HLA-ABC, and HLA-DR) were found to be undetectable (Fig. 6d). There was no significant difference in their expression when isolated using either of the isolation methods. Percentage positivity for CD73 was 8.1 ± 2% and for CD29 was 7.5 ± 3% for both UC and SUC isolated exosomes. This suggests that, during exosome biogenesis, these vesicles were most likely to be invaginated from the parental cell membrane significantly expressing CD73 and CD29. The expression of other parental cell surface markers (such as CD90 and CD105) might be there, but their expression was possibly below the detection limit.

## Discussion

Exosomes isolated from hMSCs have been explored in several studies and have been shown to mimic similar beneficial effects as hMSCs [7]. These play a significant role in cell-to-cell communications via receptor homing and also by carrying biologically active molecules like small RNA and proteins [15]. However, the above benefits and utility of the exosomes might get compromised by the low yield of exosomes by cells. To ensure a large-scale supply required for clinical application, different approaches have been evaluated and these include immortalization of producer cells like hMSCs, the use of bioreactors, or pretreatment of cells to enhance exosome production in the conditioned media [16–18]. Isolation of high-quality exosomes from hMSCs is the most crucial step to evaluate their regenerative potential and clinical use. But current strategies lack effective tools and methods for exosome isolation while maintaining the exosome integrity, high yield, and purity. Moreover, most existing strategies available with respect to exosome isolation have exploited other cell types like SK-MES-1 [8], SW480 cell line [19], and LIM1863 [20] with very limited literature available on hMSCs. Therefore, to the best of our knowledge, this is the first study that provides an in-depth comparative assessment of the two exosomal isolation protocols used in the context of hMSCs. The therapeutic value of hMSCs is evident through a number of clinical trials registered on ClinicalTrials.gov targeting a variety of diseases where hMSCs from different tissue sources (bone marrow, adipose tissue, Wharton’s jelly, and so on) have been studied [1, 2, 17, 18]. With recent advancements in mechanisms of the regenerative potential of hMSCs, the paracrine mechanism through exosomes is gaining much attention. The regenerative potential of hMSC-derived exosomes has been evaluated in various scenarios like neurodegenerative diseases, cardiovascular diseases, and kidney injury in preclinical settings. Thus, there is a need of rapid translation of these techniques for clinical application using exosomes as cell-free therapy.

Several methods available for exosome isolation have limitations such as low recovery, poor reproducibility, reduced scalability, expensive reagents and equipment, restricted production of large amounts of exosomes, or large-scale preparation of exosomes [12]. However, of all these methods, differential centrifugation is the most commonly used technique for exosomes [8]. This is due to cost-effectiveness and ease of handling large volumes of conditioned media for exosome isolation. However, Chiou et al. showed that crude exosomes which were isolated by ultracentrifugation were further purified by using sucrose gradient ultracentrifugation to separate the possible contaminants [12]. In another report, Lobb et al. compared different isolation techniques using the SK-MES-1 cell line and showed that centrifuge-based concentrating methods are more appropriate than pressure-driven concentrating devices and allow rapid isolation of exosomes. In this study, the authors compared the techniques on the basis of NTA for size and concentration of isolated exosomes and Western blotting for analyzing the resultant protein expression. With respect to NTA, ultrafiltration showed the maximum recovery of exosomes but there was no significant difference observed in protein markers for exosomes. It was also observed that repeated ultracentrifugation reduced the yield and recovery of exosomes. It was observed that, when compared with commercially available kits, current precipitation techniques perform poorly in providing pure exosome preparations [8].

Therefore, this study was planned and executed with the aim of establishing a “gold standard method” for isolation of relatively large-scale homogenous-size populations of exosomes. We used a kit-based approach for exosome isolation and characterization of protocol standardization. We used a total exosome isolation kit for cell culture in accordance with the instructions of the manufacturer (cat. no. 4478359, Thermo Fisher Scientific). However, the experiments resulted in low exosome yield (data not shown). Also, this kit could be used only for 100 mL of condition media. Therefore, we attempted to design a more effective strategy in terms of low cost and high yield of exosomes. We hypothesized that the initial loss of exosomes during the first step of differential ultracentrifugation could be prevented by a modified method which we call “one step sucrose cushion ultracentrifugation”. For this ultracentrifugation-based method, two different methods were compared: (1) standard method differential / direct ultracentrifugation (UC) and (2) a modified SUC method where samples were loaded directly onto a 30% sucrose layer for exosome isolation. Keeping sample volume constant, exosomes isolated from hMSCs by UC and SUC methods were processed further for NTA, TEM, Western blotting, and flow cytometry. For all of these techniques, the volume of samples was kept equal so that both the isolation techniques could be compared for exosome size, yield, and protein amount.

Katsuda et al. evaluated ADSC-derived exosomes for their role in Alzheimer’s disease. During this, it was observed that isolated vesicles using ultracentrifugation were mostly 175 nm in size and yield of exosomes from 1 million cells was approximately 1.5 × 10^8^ particles with a very low protein concentration of about 3 μg [21]. In contrast to this study, we have obtained exosomes with the characteristic cup shape and size ranging from 30 to 120 nm by using the SUC method. Also, using NTA, we observed that this SUC method yielded approximately 4.6 × 10^9^ particles from 5 million cells. Also, the exosomes isolated using this strategy maintained their integrity because of the cushioning effect of sucrose. This method is cost-effective and less time-consuming compared with previously used two-step sucrose gradient ultracentrifugation.

Thus, it was observed that when the starting material for exosome isolation was the same, there was a loss of exosomes when isolated using UC when compared with the one-step sucrose cushion method. Therefore, it was assumed that if we further processed these exosomes isolated by UC for second step based on sucrose cushion which is commonly used by others, the yield of exosomes would further decrease.

In this study, we have focused primarily on comparison of the standard method (UC) with the modified method (SUC) for isolation of exosomes from hMSCs, specifically ADSCs and BMSCs, for the evaluation of its yield and physical properties like morphology, size, and surface marker expression. However, the limitation of this study is the validation of this method in hMSCs derived from only two tissue types. The same method, whether extended to hMSCs derived from other tissue types such Wharton’s jelly, umbilical cord, placenta, amniotic fluid/membrane, endometrum, dental tissue, and so on, needs to be explored. The other limitation of the study is the evaluation of variation in the exosome secretion/yield from different tissue-specific hMSCs and their functional aspects using the modified method. However, our preliminary studies using exosome isolation kit, have revealed significant variation in exosome yield in tissues-pecific hMSCs (BMSCs and ADSCs) with BMSCs showing higher exosome yield within 24 h with respect to ADSCs (Additional file 1: Figure S1).. Similar results were observed in this study where BMSC-derived exosomes in conditioned media collected after 48 h yielded higher exosomes by either method in comparison with ADSCs. However, this needs to be proven by further extensive studies.

## Conclusions

Overall, we propose sucrose-based ultracentrifugation (that is, SUC) as an improved one-step method for exosome isolation from hMSC conditioned media. This method can also be used for isolation of exosomes for mass-scale production and for isolating ultrapure vesicles from other cell types for downstream protein and RNA profiling. Also, this method for isolating exosomes from hMSCs warrants further pre-clinical and clinical studies using membrane-bound exosomes with high purity.

## Additional file

Additional file 1: **Figure S1.** Characterization of hMSC-derived exosomes isolated by using a total exosome isolation kit. **a** Representative NTA graph plots depicting the number of exosomes secreted by tissue-specific hMSCs, which showed that BMSCs secrete a higher number of exosomes in comparison with ADSCs within 48 h. **b** Size distribution graph plots for hMSCs showed that, as measured by NTAs, the particle size for hMSC exosomes isolated from both BMSCs and ADSCs was within the range of 30 to 120 nm. **c** Transmission electron microscopic pictures of exosomes isolated by hMSCs showed cup-shaped morphology of exosomes. Results are mean ± standard error of the mean of three independent experiments. *Significant with *P* value of less than 0.05. Abbreviations: *ADSC* adipose tissue–derived mesenchymal stem cell, *BMSC* bone marrow–derived mesenchymal stem cell, *hMSC* human mesenchymal stem cell, *NS* non-significant, *NTA* Nanoparticle Tracking Analysis (PDF 217 kb)

## Abbreviations

ADSC: Adipose tissue–derived mesenchymal stem cell
BCA: Bicinchoninic acid assay
BMSC: Bone marrow–derived mesenchymal stem cell
BSA: Bovine serum albumin
hMSC: Human mesenchymal stem cell
NTA: Nanoparticle tracking analysis
PBS: Phosphate-buffered saline
SUC: One-step sucrose cushion ultracentrifugation
TBST: Tris-buffered saline with 0.1% Tween 20
TEM: Transmission electron microscopy
UC: Direct ultracentrifugation
