# Supplementary figures and images for: An improvised one-step sucrose cushion ultracentrifugation method for exosome isolation from culture supernatants of mesenchymal stem cells
Source: Stem Cell Res Ther. 2018 Jul 4;9:180. doi: 10.1186/s13287-018-0923-0 (PMC6033286; doi:10.1186/s13287-018-0923-0)

Fig: S1(a)

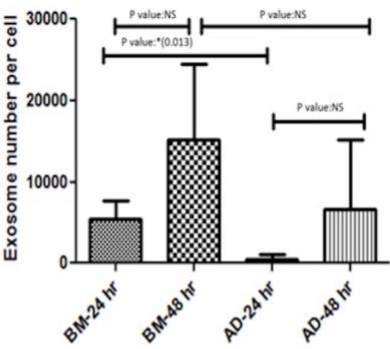

(b)

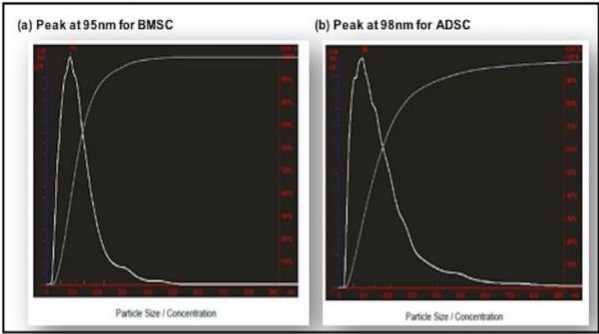

(c)

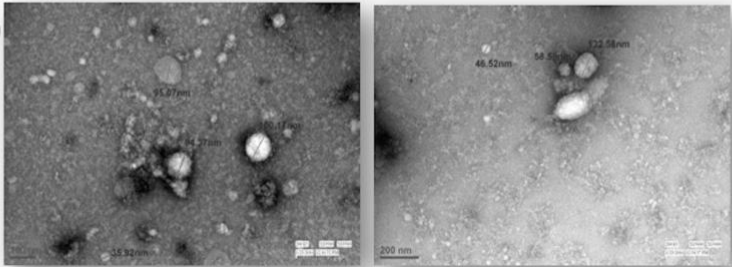

Supplement: Supplementary file 1 — Figure S1. Characterization of hMSC-derived exosomes isolated by using a total exosome isolation kit. a Representative NTA graph plots depicting the number of exosomes secreted by tissue-specific hMSCs, which showed that BMSCs secrete a higher number of exosomes in comparison with ADSCs within 48 h. b Size distribution graph plots for hMSCs showed that, as measured by NTAs, the particle size for hMSC exosomes isolated from both BMSCs and ADSCs was within the range of 30 to 120 nm. c Transmission electron microscopic pictures of exosomes isolated by hMSCs showed cup-shaped morphology of exosomes. Results are mean ± standard error of the mean of three independent experiments. *Significant with P value of less than 0.05. Abbreviations: ADSC adipose tissue–derived mesenchymal stem cell, BMSC bone marrow–derived mesenchymal stem cell, hMSC human mesenchymal stem cell, NS non-significant, NTA Nanoparticle Tracking Analysis (PDF 217 kb) [file 13287_2018_923_MOESM1_ESM.pdf]
